# Supplementary figures and images for: PRIM2 Promotes Cell Cycle and Tumor Progression in p53-Mutant Lung Cancer
Source: Cancers (Basel). 2022 Jul 11;14(14):3370. doi: 10.3390/cancers14143370 (PMC9320259; doi:10.3390/cancers14143370)

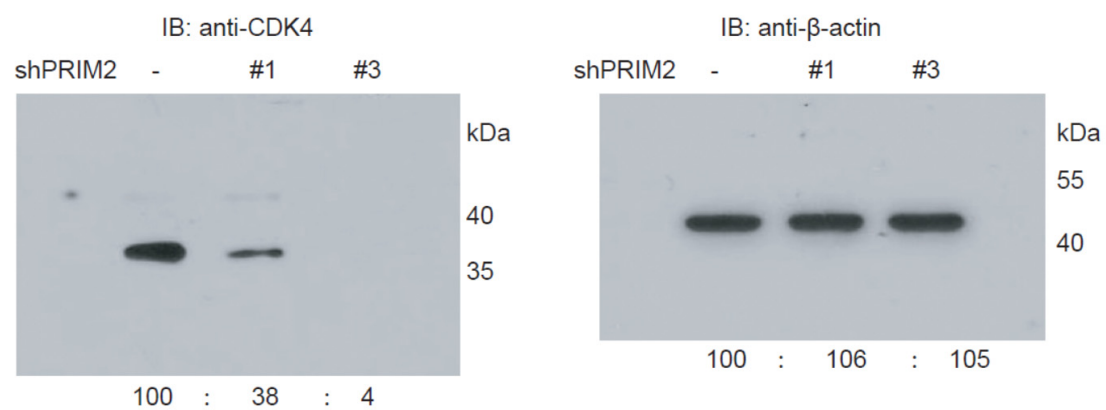

Figure S1: The uncropped WB figures is for figure 2B.

Supplement: Supplementary file 1 [file cancers-14-03370-s001.zip › cancers-1773844-supplementary.pdf]
